# Comment on “Cardiac rehabilitation after myocardial infarction: Insights into participation rates and predictors, sex differences and outcomes from a Dutch general hospital”

**DOI:** 10.1016/j.ahjo.2026.100917

**Published:** 2026-09-29

**Authors:** Yogesh Muthuraj, Sukumaran Clementswami

**Affiliations:** a Physical Education, Saveetha Institute of Medical and Technical Sciences, Chennai, Tamil Nadu, India; b Physical Education, Government Law College, Madurai, Tamil Nadu, India

**Keywords:** Pelvic floor physiotherapy, Gynecologic cancer, Cancer survivorship, Pelvic floor dysfunction, Sexual dysfunction, Vaginal stenosis, Dilator therapy, Pelvic floor muscle training, Systematic review, Rehabilitation, Treatment-related morbidity, Patient-reported outcomes

Dear Editor,

We read with great interest the study by de Fouw et al. [1], examining cardiac rehabilitation participation, sex differences, and outcomes following myocardial infarction (MI).

All-cause mortality occurred in two participants and seven non-participants, yielding a headline hazard ratio of 0.16 for the association between cardiac rehabilitation and survival [2]. A confidence interval spanning from 0.02 to 0.78 on nine total events reflects extreme statistical fragility; reclassifying even one or two deaths or a small shift in follow-up completeness between groups could move this estimate substantially or eliminate its significance entirely [3]. Presenting this hazard ratio as a headline conclusion alongside a well-powered null finding for major adverse cardiac events creates an asymmetry in how confidently these two outcomes should be read. A clinician citing this trial to counsel a patient on rehabilitation's survival benefit should understand that the point estimate, while directionally consistent with prior literature, is built on a number of events too small to exclude a substantially different true effect.

Patients who enrolled in rehabilitation were younger, more often employed, more often married, and more often treated with coronary artery bypass grafting, a pattern consistent with a cohort possessing greater baseline physiological reserve and social support rather than one rendered healthier by the program [4]. Unmeasured factors acknowledged by the authors, including left ventricular ejection fraction and medication adherence, are precisely the variables through which a sicker, more frail patient would be simultaneously less likely to complete rehabilitation and more likely to die regardless of participation. Because participation was entirely non-randomized and these variables were unavailable for adjustment, the survival association may substantially reflect who was well enough to attend rehabilitation rather than a causal effect of the program itself. The manuscript's own framing of this finding as supporting rehabilitation's benefit somewhat outpaces what an observational comparison this susceptible to healthy participant bias can establish.

Female sex predicted non-participation in the univariate analysis but not after multivariable adjustment, with the discussion attributing this to confounding by age and revascularization type. However, age at presentation and likelihood of receiving bypass surgery are not independent of sex in ischemic heart disease; women in this cohort were older at MI onset and less often revascularized surgically for reasons connected to sex-specific disease presentation and referral patterns described in the introduction's cited literature. Treating age and CABG as neutral confounders to adjust away, rather than as downstream consequences of the same sex-based disparities in cardiovascular care, the study set out to examine risks statistically removing part of the very effect under investigation rather than isolating it.

This study offers a valuable contemporary account of rehabilitation participation and confirms a persistent sex gap in completion and psychological burden that merits the targeted intervention proposed by the authors. Framing the mortality association with caution regarding its event count demands and examining whether age and revascularization type function as confounders or as mediators of sex-based disparity would allow clinicians and program designers to apply these findings with an accurate sense of which results are robust and which remain hypothesis-generating.

## CRediT authorship contribution statement

**Yogesh Muthuraj:** Conceptualization, Data curation, Formal analysis. **Sukumaran Clementswami:** Writing – review & editing, Writing – original draft, Visualization.

## Informed consent

Not applicable.

## Ethical approval

Not applicable (Commentary on a previously published article).

## Declaration of Generative AI and AI-assisted technologies in the writing process

Generative AI tools were used only for language refinement and formatting assistance. All scientific interpretation, critical appraisal, and manuscript preparation were performed independently by the authors.

## Funding

None.

## Declaration of competing interest

The authors declare that there are no conflicts of interest.

## Data Availability

No new data were generated or analyzed in this study.
